# Application of HB17, an *Arabidopsis* class II homeodomain-leucine zipper transcription factor, to regulate chloroplast number and photosynthetic capacity

**DOI:** 10.1093/jxb/ert261

**Published:** 2013-09-04

**Authors:** Graham J. Hymus, Suqin Cai, Elizabeth A. Kohl, Hans E. Holtan, Colleen M. Marion, Shiv Tiwari, Don R. Maszle, Marjorie R. Lundgren, Melissa C. Hong, Namitha Channa, Paul Loida, Rebecca Thompson, J. Philip Taylor, Elena Rice, Peter P. Repetti, Oliver J. Ratcliffe, T. Lynne Reuber, Robert A. Creelman

**Affiliations:** ^1^Mendel Biotechnology, 3935 Point Eden Way, Hayward, CA 94545, USA; ^2^Monsanto Company, 700 Chesterfield Parkway North, Chesterfield, MO 63017, USA

**Keywords:** Chloroplast number, chloroplast size, homeodomain-leucine zipper, leaf development, photosynthetic capacity, transcription factor.

## Abstract

Transcription factors are proposed as suitable targets for the control of traits such as yield or food quality in plants. This study reports the results of a functional genomics research effort that identified *ATHB17*, a transcription factor from the homeodomain-leucine zipper class II family, as a novel target for the enhancement of photosynthetic capacity. It was shown that *ATHB17* is expressed natively in the root quiescent centre (QC) from *Arabidopsis* embryos and seedlings. Analysis of the functional composition of genes differentially expressed in the QC from a knockout mutant (*athb17-1*) compared with its wild-type sibling revealed the over-representation of genes involved in auxin stimulus, embryo development, axis polarity specification, and plastid-related processes. While no other phenotypes were observed in *athb17-1* plants, overexpression of *ATHB17* produced a number of phenotypes in *Arabidopsis* including enhanced chlorophyll content. Image analysis of isolated mesophyll cells of *35S*::*ATHB17* lines revealed an increase in the number of chloroplasts per unit cell size, which is probably due to an increase in the number of proplastids per meristematic cell. Leaf physiological measurements provided evidence of improved photosynthetic capacity in *35S*::*ATHB17* lines on a per unit leaf area basis. Estimates of the capacity for ribulose-1,5-bisphosphate-saturated and -limited photosynthesis were significantly higher in *35S*::*ATHB17* lines.

## Introduction

Homeodomain-leucine zipper (HD-Zip) proteins fall into four classes (I–IV), and all contain a DNA-binding homeodomain coupled to a leucine zipper motif, which is thought to facilitate homo- or heterodimerization (Ariel *et al.*, 2007; Ciarbelli *et al.*, 2008; Elhiti and Stasolla, 2009; Harris *et al.*, 2011). HD-Zip class II proteins are implicated in mediating the response of plants to a range of environmental conditions as well as in regulating developmental processes (Ariel *et al.*, 2007; Ciarbelli *et al.*, 2008; Elhiti and Stasolla, 2009; Harris *et al.*, 2011; Ruberti *et al.*, 2012). Five HD-Zip class II genes are known to respond to changes in light quality (Ciarbelli *et al.*, 2008). For the remaining class II HD-Zip genes, little is known about their regulation other than that they are not responsive to changes in light quality (Ciarbelli *et al.*, 2008). The light responsiveness of members of the γ subclass (ATHB2, ATHAT1, and ATHAT2) has been investigated the most thoroughly. For example, when grown in white light, ATHB2 expression is induced by shading (Steindler *et al.*, 1997). ATHB2 and ATHAT1 may play roles in the shade-avoidance response, as their expression is induced by treatment with far-red-rich light, implicating a possible role for phytochrome in the regulation of these two genes (Ciarbelli *et al.*, 2008).

Light quality and intensity can also affect plastid number. For example, in plants grown under high-intensity white light, chloroplast number increases with a relative decrease in plastid size (Butterfass, 1979). Chloroplast division is a complex process that involves multiple distinct steps, which are controlled by different nuclear genes (reviewed by Pyke, 1999; Osteryoung and McAndrew, 2001; Aldridge *et al.*, 2005; Pogson and Albrecht, 2011). Higher-plant chloroplasts develop in young mesophyll cells from small plastid initials called proplastids. Proplastids are present in meristematic cells and divide in association with cell division. This division ensures that a constant proplastid number (about 14; Pyke, 1999) is maintained in all meristematic cells. As post-meristematic cells differentiate and expand, proplastids increase in size, develop into chloroplasts, and undergo approximately three rounds of division to produce the final complement of chloroplasts in a fully expanded mesophyll cell (Pyke, 1999; Aldridge *et al.*, 2005).

Here, we present results obtained with *ATHB17* (AT2G01430), which encodes a member of the α subclass of the class II HD-Zip protein family of transcription factors (Riechmann *et al.*, 2000; Ariel *et al.*, 2007; Ciarbelli *et al.*, 2008; Elhiti and Stasolla, 2009; Harris *et al.*, 2011). This study describes the expression pattern of *ATHB17* and characterizes the phenotypes of *ATHB17*-overexpressing plants and *athb17* knockout plants. The results describe the application of ATHB17 to regulate leaf morphology and chlorophyll content, the number and size of chloroplasts, and the capacity for photosynthesis in *Arabidopsis* leaves.

## Materials and methods

### Plant material and growth conditions

*ATHB17*-overexpressing lines were created in the *Arabidopsis thaliana* Heynh. ecotype Col-0. The *ATHB17* (AT2G01430) clone was PCR amplified from a diversified cDNA library created using mRNA from different *Arabidopsis* tissues. This clone contained an isoleucine substituted for methionine at position 227 and also contained 64bp of the native 5′-untranslated region (5′-UTR) immediately upstream of the start ATG and 116bp of the native 3′-UTR immediately downstream of the stop codon. *Arabidopsis* plants were transformed by the floral dip method (Bechtold *et al.*, 2003) using *Agrobacterium* carrying a transformation construct containing a kanamycin resistance gene driven by the nopaline synthase (*NOS*) promoter and the *ATHB17* clone downstream from the cauliflower mosaic virus *35S* promoter. A control line generated by transforming Col-0 with an empty transformation construct was used to determine the effect of *ATHB17* overexpression.

Between 20 and 40 independent primary transformants were isolated on selection medium and transplanted into soil. For bulk seed production, plants were grown under 24h light, in growth rooms (80–110 μmol m^–2^ s^–1^, 22 °C). Transformants were PCR genotyped to confirm that they harboured the correct transgene using forward (5′-GCAAGTGGATTGATGTGATATC-3′) and reverse (5′-TGAATTCTTCATCGTCTCCGTCTTC-3′) primers. The majority of primary transformants showed alterations in leaf shape and dark green coloration, and in some instances reductions in plant size and alterations in inflorescence morphology were apparent. Expression of *ATHB17* mRNA transcripts was verified by reverse transcription-PCR (RT-PCR) on RNA extracted from leaves of 40-d-old plants (see below). Lines that showed overexpression of *ATHB17* by RT-PCR versus the control line were selected and used in subsequent experiments.

For morphological analysis and photosynthetic measurements, seeds were surface sterilized and stratified at 4 °C for 3 d. Seeds were sown on 80% Murashige and Skoog (MS) medium plus vitamins, 1% sucrose and kanamycin (35mg l^–1^) and placed in a growth chamber (ATC 26, Controlled Environments; photosynthetic photon flux (PPF) of approximately 120–150 μmol m^–2^ s^–1^; 10 h:14h light:dark; 22 °C:19 °C day:night). After 6–10 d of growth on selection medium, seedlings were transplanted into autoclaved Promix soil and grown in an ATC 26growth chamber as above. After 1 week of growth on soil, plants were given weekly fertilizer treatments with 0.4 or 0.8g l^–1^ of Peter’s fertilizer. Plants were about 30–42 d old when used for experiments.

To examine the endogenous expression of *ATHB17* transcripts by *in situ* RNA–RNA hybridization, seeds were prepared as above and germinated on solid 80% MS medium (80% MS plus vitamins, 0.3% sucrose, and 1% BactoAgar) with a 16 h:8h light:dark photoperiod for 6 d. Seedlings were then transferred to vertical plates and grown for an additional 6 d. Seedlings that were 12 d old were used for *in situ* analysis of shoot apices and roots. Soil-grown plants were used to analyse the later stages of *Arabidopsis* growth. Seeds were germinated directly in soil after 2 d of stratification in the dark at 4 °C. Plants were grown in a 16 h:8h light:dark photoperiod at 22 °C in a growth chamber. Inflorescence, floral meristems, and developing seeds were collected from 4–5-week-old plants.

For laser-capture microdissection, 11-d-old seedlings (grown as above for *in situ* analysis) from *athb17-1* and its wild-type sibling were used for collection of specific root tissues.

### RNA isolation and quantitative RT-PCR (qRT-PCR)

Expression of the *ATHB17* mRNA transcripts in *athb17* mutant and overexpressing plants was examined by qRT-PCR. RNA was isolated from frozen root tips or 14-day-old seedlings using a Qiagen RNeasy Plant Mini kit and reverse transcribed (Superscript II RT; Invitrogen) using a mixture of oligo(dT) and random hexamer primers (60 and 40% respectively). cDNA templates were then analysed by real-time PCR (7900 HT Fast Real-Time PCR; Applied Biosystems) using a SYBR Green PCR Mix (Applied Biosystems).

Primers used for the RT-PCR were: *ATHB17* transformation construct (5′-TTCGTGTAAATACTAAGAGACTCTGTTCCG-3′ and 5′-TGCCATAATACTCAAACTCAGTAGGA-3′), *ATHB17* native expression (5′-AGGTGGTTTGGTTCATTAACGGAAG-3′ and 5′-GCAGCGAGGACACATAGTAAGGC-3′), *ATHB2* (5′-ACATG AGCCCACCCACTACTTTGAC-3′ and 5′-CAGGAGCCCACGC ATTGACC-3′), *ATHAT2* (5′-AGCACAATACTCTCAATCCCA-3′ and 5′-CTGCTTTAACTTTGTCCTTGCTC3′), *ATHAT1* (5′-GAA CACAACACTCTCAATCCC-3′ and 5′-TTCAAGTATTCGCAAT CTACCTCC-3′) and a mid-level constitutive control gene *AT2G32170* (5′-GCAGGAATAACAGAAGGTTTCTCTATGTGT-3′ and 5′-AT GAGCAGTGTCGATGAAAAAGCA-3′; Czechowski *et al.*, 2005). Expression levels were calculated based on Δ*C*_t_, the difference in cycle threshold (*C*_t_) between the gene of interest and the control, with signals normalized to *AT2G32170*, a mid-level constitutive control gene.

### Protoplast-based transcriptional repression assay

The cDNA sequence encoding the ATHB17 protein was cloned downstream of the cauliflower mosaic virus *35S* promoter and a translational enhancer from the 5′-leader of tobacco mosaic virus (Skuzeski *et al.*, 1990). All promoter sequences were PCR amplified and cloned upstream of the *GUS* reporter gene. The *Agrobacterium NOS* transcriptional terminator was used in all reporter and effector plasmids (Skuzeski *et al.*, 1990). The sequence length of each promoter was as follows: *ATHAT1* (1821bp), *ATHB2* (1500bp), and *AGL8* (1833bp). The nucleotide position of each promoter was the number of bases upstream of the translation start site of the respective genes. The *35S*::*CAT* reporter gene construct encoding chloramphenicol acetyltransferase (CAT) has been described previously (Tiwari *et al.*, 2006).

Protoplasts were prepared from leaves of 3–4-week-old *Arabidopsis* plants using cellulase (Research Products International) and macerozyme (SERVA Electrophoresis; Tiwari *et al.*, 2006). For the assay, 200 μl of protoplast cells was co-transfected with 10 μg of the *ATHAT1*::*GUS*, *ATHB2*::*GUS*, or *AGL8*::*GUS* reporter gene construct and either 10 μg of the *35S*::*CAT* control construct or a *35S*::*ATHB17* construct. Plasmid DNA (EndoFree Plasmid Maxi; Qiagen) was introduced into protoplasts by incubating with 40% polyethylene glycol for 30min. After polyethylene glycol removal, protoplasts were kept in darkness at room temperature for 18–20h. Cells were lysed and incubated with 1mM 4-methylumbelliferyl-β-d-glucuronide (Gold Biotechnology) at 37 °C for 1h. β-Glucuronidase (GUS) activity was quantified by taking fluorescence measurements using a Synergy HT Microplate Reader (BioTek). GUS measurements for each construct were averaged (*n*=3).

### In situ RNA–RNA hybridization

DNA templates were generated by PCR using gene-specific primers (*ATHB17*-forward, 5′-CTTCGATCCTAGCTCTTAAGAACC-3′, and *ATHB17*-reverse, 5′- GTAACCGATTCATGTCTAGCC-3′) with incorporation of a T7 promoter sequence (5′-CTAATACGACTCA CTATAGGG-3′) on one primer (Baklanov *et al.*, 1996; Iacobuzio-Donahue *et al.*, 2002). To generate template for the sense and antisense probes, the T7 promoter sequence was incorporated at the 5′ end of *ATHB17*-forward and the 5′ end of *ATHB17*-reverse, respectively.

Digoxigenin (DIG)-labelled RNA probes were generated by *in vitro* transcription using T7 RNA polymerase (Life Technologies) except that a 1:2 ratio of UTP:DIG-11-UTP (Roche Applied Science) was used during the reaction. Fixation of tissues, preparation of sections, hybridization, and washes were carried out as described by Jeffrey Long (http://www.its.caltech.edu/~plantlab/protocols/insitu.pdf, last accessed 26 July 2013) with the following modifications: (i) RNAase treatment was omitted; (ii) the blocking reagent was 1% blocking reagent in 1× maleic acid buffer (Roche Applied Science); (iii) the wash buffer contained 1× maleic acid buffer with 0.03–0.05% Tween 20 (v/v) (Roche Applied Science); and (iv) sections were mounted using Immu-Mount (Thermo Scientific).

### Preparation of paraffin sections and laser-capture microdissection

Primary and lateral root tips from 11-d-old seedlings were used for the collection of specific root tissues. Preparation of paraffin-embedded tissues (Kerk *et al.*, 2003; Jiao *et al.*, 2009) was performed with minor modifications. Specifically, root tips were fixed in ice-cold fixative (70% ethanol, 15% acetic acid, 0.6% trehalose) for approximately 3h, and then treated twice with 75% alcohol for 30min each and 90% alcohol for 45min. Stepwise infiltration of paraffin was done with a TP1020 automatic tissue processor (Leica Microsystems): 100% ethanol for 1h, 0.2% Eosin Y (in 100% ethanol) for 30min, 100% ethanol for 2h, ethanol/Histo-Clear series (3:1, 1:1, 1:3, 0:1, 0:1, 1–2h each), and paraffin (for 4 and 8h with vacuum). Paraffin sections (8 μm) were prepared as described previously (Jiao *et al.*, 2009).

Paraffin sections were deparaffinized in Histo-Clear and air dried before slides were viewed using the fluorescence mode and the rhodamine reflector of a PALM MicroBeam System (Carl Zeiss) to facilitate the identification of different cell types. The root cap, quiescent centre (QC), epidermis/endodermis/cortex, and stele tissues were collected into adhesive caps (Carl Zeiss). Three biological replicates, each pooled from 40–60 roots, were collected for each sample

### Microarray analysis

Total RNA was prepared and amplified and cRNA was generated from microdissected tissues using commercial kits (PicroPure RNA, RiboAmp HS RNA, and Turbo Labeling Biotin kits, respectively; Life Technologies). Transcript profiling experiments were performed using a custom full-genome *Arabidopsis* Affymetrix GeneChip (mbiATh1a520184) microarray and analysed according to Holtan *et al.* (2011). Calculations for the over-representation analysis were performed according to Holtan *et al.* (2011).

The Affymetrix data files (.CEL), pre-processed profiles, and supplementary files described herein have been deposited at the National Center for Biotechnology Information (http://www.ncbi.nlm.nih.gov/geo, last accessed 26 July 2013) under GEO accession no. GSE48127.

### Preparation and analysis of mesophyll cell suspensions for chloroplast counting

Chloroplasts were counted using Nomarski differential interference contrast optics (Leica DMR-HC; Leica Microsystems) in individual fixed mesophyll cells obtained by the maceration of prepared leaf tissue (Pyke and Leech, 1991).

### Estimating biochemical limitations to light-saturated photosynthesis

The protocol of Long and Bernacchi (2003) guided the generation of plots of light-saturated photosynthesis (*A*_sat_) and the rate of linear electron transport through photosystem II (*J*_PSII_) to substomatal [CO_2_] (*C*_i_) for the youngest, fully expanded leaves of plants for a control line and two *35S*::*ATHB17* lines using an infra-red gas analyser with a modulated chlorophyll *a* fluorimeter (LI-6400 XT; Licor). Initial measurements were made at an atmospheric [CO_2_] (*C*_a_) of 400 μmol of CO_2_ mol^–1^, after 40min exposure to a PPF of 700 μmol m^–2^ s^–1^. After the initial measurement, further measurements were made after three stepwise decreases in *C*_a_ to 150, 100, and 50 μmol of CO_2_ mol^–1^. After a second measurement at 400 μmol of CO_2_ mol^–1^, three final measurements were made after stepwise increases in *C*_a_ to 600, 700, and 800 μmol of CO_2_ mol^–1^. Measurements for all three lines were made at the same leaf–air water vapour pressure deficit of about 1 kPa and relative humidity of about 50%.

The curve-fitting software of Sharkey *et al.* (2007) and kinetic constants described therein were used to make *in vivo* estimates of: (i) the maximum rate of ribulose-1,5-bisphosphate (RuBP)-saturated photosynthesis (*V*_c,max_), from the three measurements of *A*_sat_ and *C*_i_ made at the lowest *C*_a_ levels; and (ii) the light-saturated capacity for RuBP regeneration, calculated and expressed in terms of electron flow required to support the modelled rate of *A*_sat_ (*J*_max_), from the three highest C_a_ levels. Estimates of *J*_PSII_ were estimated as the product of the operating efficiency of PSII and the fraction of incident PAR absorbed by PSII (Genty *et al.* 1989). The fraction of the 700 μmol m^–2^ s^–1^ of PAR incident upon the leaf in the leaf chamber that was transmitted through leaves was measured by pressing a quantum sensor (LI 190; Licor) to the centre of the abaxial side of the leaf. No measure of reflectance from the adaxial leaf surface was made; consequently absorption was calculated as 1 – transmission for the determination of *J*_PSII_. We assumed that 50% of incident PAR was absorbed by PSII for the purposes of calculating *J*_PSII_.

### Chlorophyll assays

Chlorophyll content was measured using a SPAD 502 (Konica Minolta).

### Data analysis

Mesophyll cell plan areas, chloroplast number per cell, and individual chloroplast plan areas were measured directly from the microscope image using ImageJ (Abramoff *et al.*, 2004). Data from leaves were averaged to give a mean and standard error (SE). Significance was tested with a Student’s *t*-test and a standard linear regression model using R (version 2.2.0; R Development Core Team, 2007).

The hypothesis that *35S*::*ATHB17* plants had increased *V*_c,max_ and *J*_max_ compared with the empty-vector control plants was tested with a one-tailed Student’s *t*-test using Excel (Microsoft). Data analysis was performed on six replicate samples unless otherwise stated and an effect was described as statistically significant when *P* < 0.05.

## Results and discussion

### ATHB17 is expressed in the QC of the primary and lateral roots

**Fig. 1.**
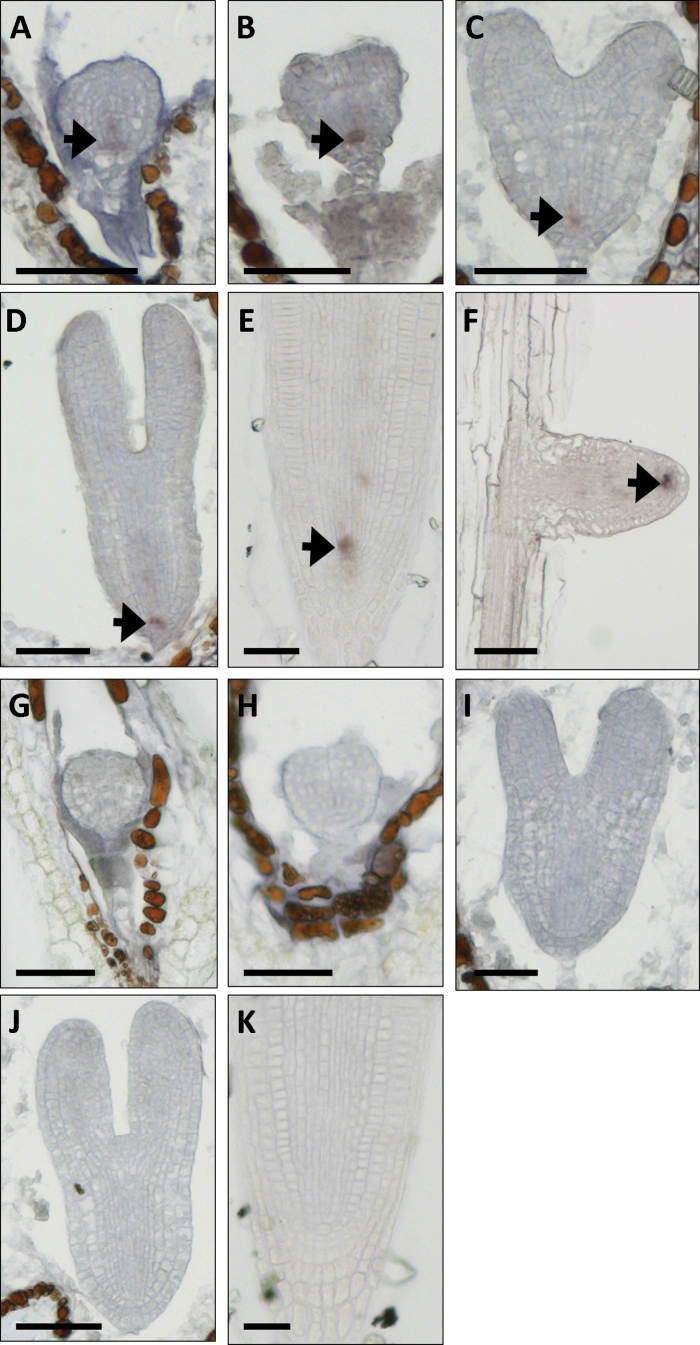
*In situ* hybridization using DIG-labelled antisense and sense probe targeting nt 173–353 of the *ATHB17* coding sequence. (A–F) Antisense probe, (G–K) sense probe. (A–D, G–J) Embryos at the globular stage (A, G), transition stage (B, H), heart stage (C, I), and linear cotyledon stage (D, J). (E, K) Primary root tip. (F) Lateral root tip. In each panel, the region of greatest hybridization signal is highlighted with a black arrow. Bars, 50 μm.

We analysed the spatial expression pattern of *ATHB17* by *in situ* RNA–RNA hybridization (Fig. 1A–F) using DIG-labelled antisense and sense probes targeted to a unique region of the *ATHB17* coding sequence (nt 173–353). Of the tissues examined (roots, shoot apical meristems, inflorescence meristems, floral meristems, and developing embryos), hybridization was only detected in specific cells in embryonic, primary, and lateral roots. In a survey across a developmental series, *ATHB17* signal was first detected in the base of globular stage embryos, and was then restricted at the transition stage to the lens-shaped cell, which gives rise to the QC. In serial sections from older embryos and the primary root tip of seedlings (Fig. 1A–E) as well as the tips of lateral roots (Fig. 1F), *ATHB17* expression continued to be localized specifically to the small number of cells that comprised the QC. No signal was detected using the control sense probe (Fig. 1G–K).

The root is comprised of several tissues that undergo predictable divisions. Initials for all root tissues surround a group of four to seven mitotically less active cells known as the QC. The QC is important for proper root development and regulates processes and events in neighbouring cells for proper meristem establishment and maintenance (Doerner, 1998; Jiang and Feldman, 2005). The root QC-specific expression of *ATHB17* implied that *ATHB17* plays a role in the regulation of root growth and development.

### Comparison of root QC transcript profiles between athb17-1 and its wild-type sibling

We screened T-DNA insertion lines (Alonso *et al.*, 2003) and identified three different *ATHB17* alleles (Supplementary Fig. S1 at *JXB* online). RT-PCR analysis indicated a significant reduction of the normal *ATHB17* transcript in the line designated *athb17-1*, containing a T-DNA insertion of 162bp into the third intron of *ATHB17* (Supplementary Fig. S2A at *JXB* online). Western blot analysis confirmed the absence of ATHB17 in the root tips of *athb17-1*, but not in *athb17-2* and *athb17-3* (Supplementary Fig. S2B). The *athb17-1* line was used for further analysis.

We examined how *ATHB17* regulates gene expression by transcript profiling of *athb17-1* and its wild-type sibling. To increase our sensitivity to effects caused by the loss of ATHB17, we analysed tissue samples (collected by laser-capture microdissection) that were highly enriched with QC cells. To validate that we were able to enrich for QC cells by laser microdissection, a small panel of genes was selected and their transcript levels analysed by qRT-PCR across four distinct tissue types dissected from the root tip: QC, root cap, stele, and epidermis. The expression of known QC-localized genes [*PLETHORA 1* (*PLT1*), *WIP4*, and *ATMYB34;* Aida *et al.*, 2004; Nawy *et al.*, 2005] was enriched in QC samples (Supplementary Fig. S3A at *JXB* online). On the other hand, genes known to be expressed in stele (AT5G25490; Nawy *et al.*, 2005) or root cap tissue (*ATMDK-20;* del Campillo *et al.*, 2004; Lilley *et al.*, 2011) displayed stele- or root cap-enriched expression, respectively (Supplementary Fig. S3B, C).

**Fig. 2.**
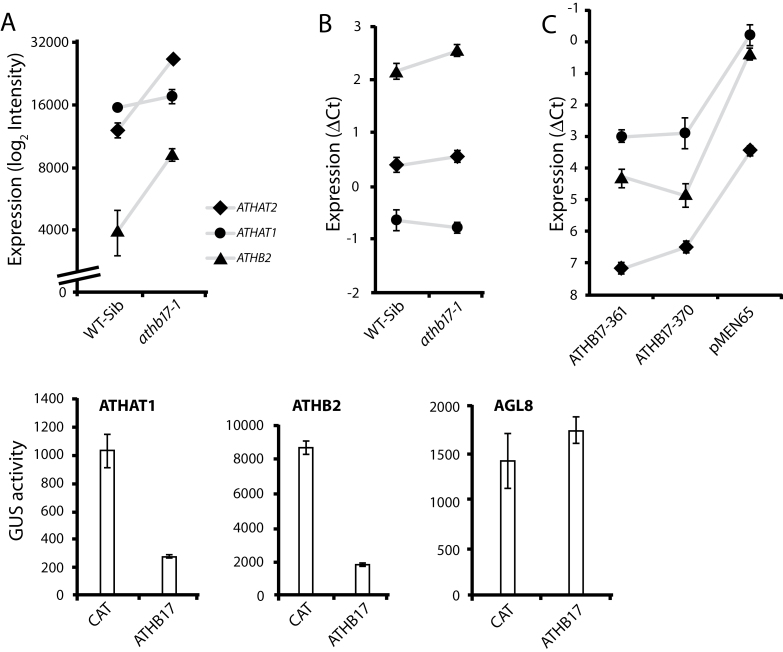
Expression levels of *ATHAT2*, *ATHAT1*, and *ATHB2* in various *Arabidopsis* tissues. Microarray analysis indicated that the transcript levels of selected class II HB-Zip genes were altered in the QC-enriched samples of *athb17-1* compared with its wild-type sibling. (A) *ATHAT2* and *ATHB2* expression, but not *ATHAT1* expression, was induced in the *athb17-1* QC. (B) qRT-PCR was used to verify the altered expression pattern observed in panel A. (C) *ATHAT1*, *ATHAT2*, and *ATHB2* expression is significantly repressed in seedlings of two *35S*::*ATHB17* lines (line 361 and line 370). (D) ATHB17 also repressed the transcription of *ATHAT1* and *ATHB2* in *Arabidopsis* mesophyll protoplasts. Full-length *ATHB17* or *CAT* was co-transfected with the *pATHAT1*::*GUS* or *pATHB2*::*GUS* reporter gene into *Arabidopsis* mesophyll protoplasts. The *pAGL8*::*GUS* reporter gene was used as a non-specific reporter as it was not expected to be regulated by ATHB17. The data are means ±standard deviation (SD) of three replicates. PCR comparisons were made after normalization to a constitutive control gene by the ΔCt method.

Of the three class II HD-Zip genes expressed in the root QC (Nawy *et al.*, 2005), *ATHB2* and *ATHAT2* showed increased expression in the QC of *athb17-1* roots relative to the wild-type control, while *ATHAT1* did not (Fig. 2A). We confirmed the effect on *ATHB2* and lack of effect on *ATHAT1* by performing qRT-PCR using RNA isolated from whole root tips of *athb17-1* and its wild-type sibling (Fig. 2B). The more subtle effect on *ATHAT2* observed in the array experiments could not be confirmed in whole root tips. The array and qRT-PCR results suggested that ATHB17 functions to repress the expression of *ATHB2* and *ATHAT2* in the QC, or that ATHB17 may act to repress an activator of these genes. Indeed, in seedlings of *35S*::*ATHB17* lines in which *ATHB17* was constitutively expressed at high levels, *ATHB2*, *ATHAT1*, and *ATHAT2* were all strongly repressed (Fig. 2C). We tested whether ATHB17 could regulate the expression of *ATHB2* and *ATHAT1* using a transient expression assay. We co-transfected constructs encoding *ATHB17* or *CAT* and *pATHB2*::*GUS* or *pATHAT1*:*GUS* reporter genes (Fig. 2D) into *Arabidopsis* mesophyll protoplasts. The *pAGL8*::*GUS* reporter gene was used as a non-specific reporter that was not expected to be regulated by ATHB17 protein. The transcription of the reporter gene driven by either class II HD-Zip promoter was strongly repressed in the presence of ATHB17. Thus, class II HD-Zip gene expression within the root QC was regulated by other class II HD-Zip proteins and may function as a negative regulatory loop in which homeostasis is mutually controlled (Ariel *et al.*, 2007; Sorin *et al.*, 2009; Harris *et al.*, 2011). The specific expression of *ATHB17* in the root QC suggested that ATHB17 may be involved in some aspects of root development. However, the roles that ATHB2, ATHAT1, ATHAT2, and ATHB17 play in affecting root growth are unknown at this time.

### Several related biological processes are subtly perturbed in athb17-1

**Fig. 3.**
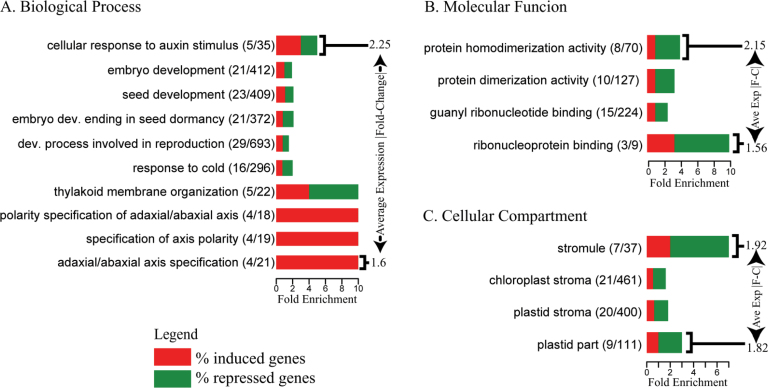
Functional category analysis of genes differentially expressed in the root QC of *athb17-1* compared with the wild-type sibling line. Over-representation of terms from different aspects of GO, including (A) *Biological Process*, (B) *Molecular Function*, and (C) *Cellular Compartment* are shown separately. Based on analysis of all GO terms annotated to *Arabidopsis* genes (Berardini *et al.*, 2004), the categories listed above were over-represented (*P* <0.01) among significantly mis-expressed genes. For each over-represented GO category, we have shown the number of genes with significantly altered expression and the total number of genes annotated to the term. The horizontal length of the bar indicates the ratio of the number of genes annotated to the term that have significant expression changes relative to the number of genes from that category that would have been expected to show such changes by chance, while the vertical length of the bar represents the magnitude of the average absolute fold change of these genes. The percentage of induced and repressed genes annotated to each GO term is indicated by red and green, respectively. See Supplementary Table S1 at *JXB* online for a list of genes over-represented in each of the GO categories.

Gene set over-representation analysis using gene ontology (GO) terms was used to determine which biological processes and molecular functions showed the greatest differences between *athb17-1* and its wild-type sibling (Fig. 3). Genes differentially expressed between *athb17-1* and its wild-type sibling with annotation to biological processes that included responses to auxin stimulus, embryo development, and axis polarity were over-represented. Functional analyses indicate that some HD-Zip class II genes (*ATHB2*, *ATHB4*, and *ATHAT2*) are linked to auxin (Steindler *et al.*, 1999; Sawa *et al.*, 2002), consistent with our observations on the expression of class II HD-Zip genes in *athb17-1* QC cells.

The presence of genes in GO categories related to embryo or seed development and axis polarity (Fig. 3) was interesting considering that, while class II HD-Zips are implicated in the integration of shade-avoidance responses and hormone-mediated growth (Sawa *et al.*, 2002; Sorin *et al.*, 2009), class III HD-Zip proteins and KANADI are characterized as developmental regulators during embryogenesis, the establishment of the apical meristem, lateral root formation, and the determination of leaf polarity (Hawker and Bowman, 2004; Prigge *et al.*, 2005; Izhaki and Bowman, 2007). The expression of *PHAVOLUTA* (AT1G30490), *KANADI 2* (AT1G32240), and *REVOLUTA* (AT5G60690) was induced in the QC of *athb17-1* roots (Supplementary Table S2 at *JXB* online). A common link between class II/III HD-Zip proteins and KANADI-related proteins is auxin. Auxin concentration and flux mediated by PIN-FORMED1 (PIN1) contribute to embryo patterning during embryogenesis and the establishment of the apical–basal axis and bilateral symmetry (Izhaki and Bowman, 2007).

A number of genes with GO terms specifying plastid-related processes and constituent parts including thylakoid membrane organization, stroma, stromule (Kohler and Hanson, 2000), and the term ‘plastid part’ were significantly over-represented, with more genes repressed than induced in the *athb17-1* mutant.

### A whole-plant phenotype comparison among wild-type, 35S::ATHB17, and athb17-1 Arabidopsis plants

The *athb17-1* plants displayed no clear differences from wild-type controls when grown on plates to examine potential root phenotypes or when grown in soil to examine overall morphology and chlorophyll content (Supplementary Fig. S4 at *JXB* online), which suggested that ATHB17 is functionally redundant with other class II HD-Zip proteins in the root QC. The primary and branch root lengths as well as branch root number were similar in both *athb17-1* and its wild-type sibling (Supplementary Fig. S4A–C). No detectable rosette phenotype was observed (Supplementary Fig. S4D, E). On the other hand, *athb17-1* may produce a conditional phenotype or our experimental growth conditions may not have been sensitive enough to detect a discernible phenotype.

**Fig. 4.**
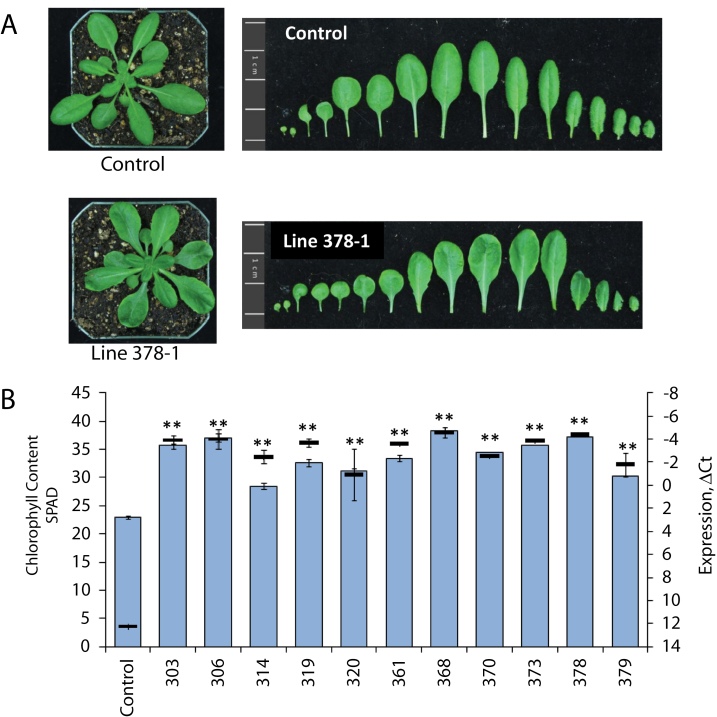
(A) Plants (30 d old) grown under short-day conditions illustrating that the transgenic plants (T3) had leaves that were darker green, and rounder with a slight leaf blade on petiole phenotype.(B) A high correlation was found between chlorophyll content determined by SPAD (blue) and *ATHB17* transgene expression (black, shown relative to the constitutive control gene *AT2G32170*) was demonstrated across 11 T2 *35S*::*ATHB17* lines. Results are shown as the mean±SE (*n*=20). **P <0.05.

In contrast, overexpression of *ATHB17* produced marked phenotypic effects; in particular, the leaves of the transgenic lines were consistently visibly darker green (Fig. 4A). A series of *35S*::*ATHB17* lines was examined for this phenotype; chlorophyll content as measured by soil plant analysis development (SPAD) was higher in all lines (Fig. 4B) and was positively correlated with *ATHB17* mRNA levels. Chlorophyll levels in leaves as measured by SPAD from *athb17-1* plants were comparable to those seen in empty-vector control leaves (Supplementary Fig. S4F). Leaf blade development in *35S*::*ATHB17* lines was also de-repressed along the petiole, creating a loss of distinct proximal and distal zones and resulting in a slight leaf blade on petiole phenotype that resulted in a rounder appearance (Fig. 4A; Ha *et al.*, 2003; Hepworth *et al.*, 2005). We have suggested that, within the root QC, ATHB17 with ATHB2, ATHAT1, and ATHAT2 form a negative regulatory loop. It has been widely demonstrated that the expression of class II HD-Zip genes is mutually regulated, in that when one class II HD-ZIP gene is overexpressed, the expression of other class II HD-Zip genes is repressed (Ohgishi *et al.*, 2001; Sawa *et al.*, 2002; Sorin *et al.*, 2009; Harris *et al.*, 2011). The phenotypes observed with constitutive overexpression of *ATHB17* could result from repression of other class II HD-Zip genes or other native targets during embryo development and growth during the seedling and rosette stages. The slight blade on petiole phenotype (Ha *et al.*, 2003; Hepworth *et al.*, 2005) suggested that subtle alterations in leaf development are caused by *ATHB17* overexpression. Leaf pattern formation is dependent on the establishment of the abaxial/adaxial axis because loss of either abaxial or adaxial identity perturbs leaf blade outgrowth and symmetry. Consequently, some leaf phenotypes in *35S*::*ATHB17* plants may result from perturbations in abaxial/adaxial polarity mediated by effects on auxin transport/signalling or the disruption of a complex regulatory network consisting of various class II and class III HD-Zips plus KANADI-like proteins (Harris *et al.*, 2011; Brandt *et al.*, 2012).

### Chloroplast and cell characteristics of 35S::ATHB17 plants

**Fig. 5.**
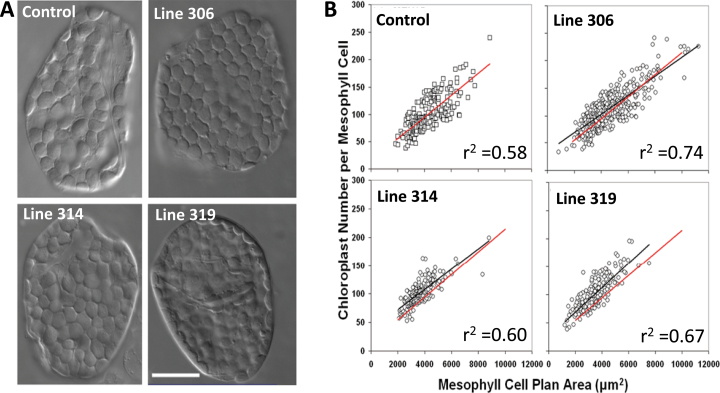
Isolated leaf mesophyll cells (A) and chloroplast number per mesophyll cell versus cell size (B) from the youngest fully expanded leaves of 40-d-old control or *35S*::*ATHB17* lines. (A) Mesophyll cells were viewed with Nomarski differential interference contrast optics. Bar, 25 μm. (B) Each data point represents the measurement from one cell. The solid red line in each figure represents the regression line for the empty-vector control line, while the solid black line is the regression of the transgenic in each panel. Values for *r* ^2^ are indicated on the graphs.

Representative cellular phenotypes of typical mature leaf mesophyll cells of an empty-vector control line and three *35S*::*ATHB17* lines (306, 314, and 319; T2 generation) are illustrated in Fig. 5A. Control and *35S*::*ATHB17*-overexpressing lines showed a close relationship between plastid number and mesophyll cell size (Fig. 5B). *35S*::*ATHB17* plants accumulated more plastids per unit cell area compared with the control line (Table 1). The distribution of chloroplast plan area in fully expanded mesophyll cells was also investigated and is shown in Table 1. *35S*::*ATHB17* lines exhibited a slight reduction in plastid plan area compared with the control line. The proportion of mesophyll surface area covered by chloroplasts was consistent between *35S*::*ATHB17*-overexpressing lines and the control line (data not shown). This may result from compensation between chloroplast number and chloroplast size; i.e. *35S*::*ATHB17* mesophyll cells have more chloroplasts per cell than a wild-type control line, but the chloroplasts are somewhat smaller.

**Table 1.** *Mean chloroplast plan area, mean mesophyll plan area, and plastid number for populations of mesophyll cells from the youngest fully expanded leaves of 40-d-old control and* 35S*::*ATHB17 Arabidopsis *plants*Chloroplast plan areas were measured by image analysis of fixed isolated cells from three *35S*::*ATHB17*-overexpressing lines (T2 generation) and an empty-vector control line (pMEN65) in two independent experiments. Plastid number per cell (±SE) was determined from a regression line of chloroplast number per cell on mesophyll cell plan area using a value of 1000 μm^2^ for the mesophyll cell plan area (Fig. 5). The *y*-intercept of the regression line was used to estimate the number of proplastids (±SE) in very small, meristematic cells. For Experiments 1 and 2, mean chloroplast plan area is the mean ±SE of 600 chloroplasts from 30 or 60 different mesophyll cells, respectively. **P <0.05.

Genotype	Mean chloroplast plan area (μm^2^)	Plastids	
		No. chloroplasts per 1000 μm^2^ mesophyll cell plan area	Estimated number proplastids per meristematic cell
Experiment 1			
pMEN65	39.8±0.4	35±2	14±2
*35S*::*ATHB17* line 306	35.9±0.3**	51±1**	33±1**
*35S*::*ATHB17* line 314	35.0±0.3**	55±1**	37±1**
*35S*::*ATHB17* line 319	38.3±0.4**	48±1**	25±1**
Experiment 2			
pMEN65	36.7±0.4	44±1	22±2
*35S*::*ATHB17* line 306	32.7±0.3**	70±1**	48±1**
*35S*::*ATHB17* line 314	32.7±0.3**	67±1**	44±1**
*35S*::*ATHB17* line 319	30.5±0.4**	57±1**	34±1**

Based on published data (Pyke and Leech, 1992; Pyke, 1997), the smallest mesophyll cells (500–800 μm^2^, representative of post-meristematic cells) of fully expanded mature leaves have approximately 10–20 chloroplasts. However, based on our regression analysis (Fig. 5B), the number of plastids in the smallest cells of *35S*::*ATHB17* plants was greater than that found in the youngest cells of an empty-vector control line. Therefore, the number of proplastids per meristematic cell is potentially greater in *35S*::*ATHB17* plants than in controls (Table 1). These data suggest that *ATHB17* overexpression regulates proplastid division rather than chloroplast division.

### Light-saturated photosynthetic capacity is increased in 35S::ATHB17 Arabidopsis plants

**Fig. 6.**
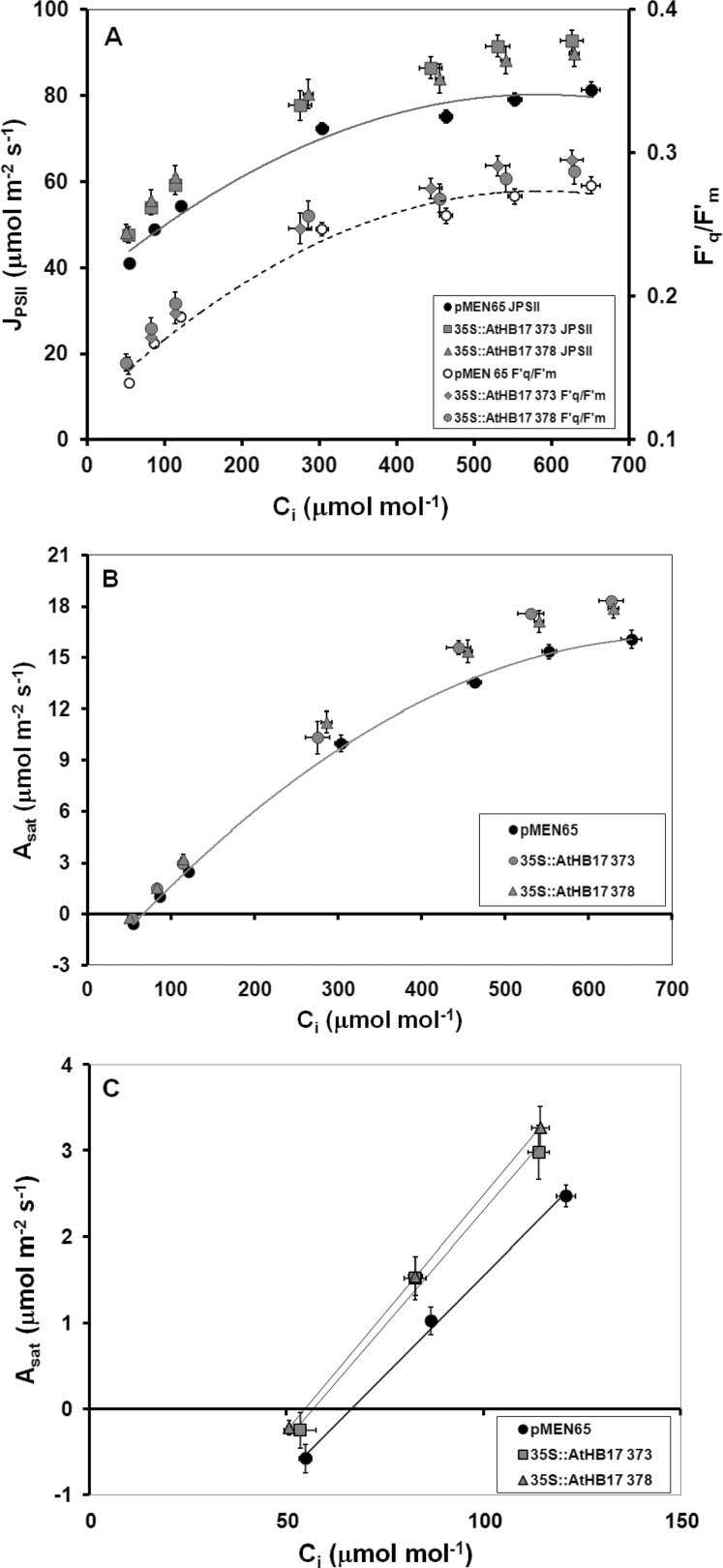
Plot of *J*_PSII_ and *F*′_v_/*F*′_m_ (A) and *A*_sat_ (B, C) against *C*_i_ for select 38-d-old *35S*::*ATHB17* lines and an empty-vector control line (pMEN65). The data shown in (C) give an expanded view of the CO_2_-limited part of the larger *A*_sat_/*C*_i_ response curve shown in (B). The photosynthetic phenotypes reported have proved to be highly penetrant across multiple *35S*::*ATHB17* lines, and the effects described were repeatable in multiple independent experiments. Results are shown as the mean±SE (*n*=6) from a representative experiment.

Two well-characterized *35S*::*ATHB17* lines (373-3 and 378-1; T3 generation) were studied to investigate whether increases in leaf chlorophyll content would impact photosynthesis in *Arabidopsis*. For both *35S*::*ATHB17* lines, leaf SPAD was significantly increased, by 36% relative to the control line (Table 2). Increased leaf chlorophyll was consistent with a significant 6% decrease in transmission of PAR through the leaves in the *35S*::*ATHB17* lines (Table 2). The decrease in transmission of PAR through the *35S*::*ATHB17* leaves was probably due to increased PAR absorption, an effect that could be reconciled with a significant increase in *J*_PSII_ of up to 14% in the two *35S*::*ATHB17* lines, relative to the control line, over a range of *C*_i_ (Fig. 6A). This increase in *J*_PSII_ was largely the consequence of increased light absorption, with increases in the operating efficiency of PSII (*F*′_q_/*F*′_m_) in the *35S*::*ATHB17* lines typically being smaller over the same range of *C*_i_ (Fig. 6A).

**Table 2.** *Key components of photosynthetic capacity are increased in* 35S*::*ATHB17 *lines*Sample means ±SE are shown for leaf SPAD, percentage transmission of PAR, and estimates of *V*_c,max_ and *J*_max_. Data were collected on at least five replicate plants for the empty-vector control and two *35S*::*ATHB17* lines. The means of each data set collected for the two *35S*::*ATHB17* lines were significantly different from the control line (*P* <0.05; two-tailed Student’s *t*-test).

Genotype	SPAD	Transmission (%)	*V*_c,max_	*J*_max_
pMEN65	31.2±0.5	12.7±0.5	28.8±1.1	80.4±2.3
*35S*::*ATHB17* line 373-3	43.9±2.0	5.4±0.5	33.1±1.6	90.2±2.3
*35S*::*ATHB17* line 378-1	42.4±1.4	5.8±0.4	33.6±2.1	87.5±2.7

The plots of *J*_PSII_ and *A*_sat_ (Fig. 6B) against increasing *C*_i_ provided evidence of increased photosynthetic capacity in both *35S*::*ATHB17* lines. A progressive stimulation of *A*_sat_ relative to the control line was seen as *C*_i_ increased in both *35S*::*ATHB17* lines (Fig. 6B). AT the three highest C_i_ values, both *A*_sat_ and *J*_PSII_ were significantly higher in the *35S*::*ATHB17* lines; however, while *A*_sat_ was still rising in all lines, *J*_PSII_ was reaching, or had reached, a plateau. Increasing *A*_sat_ over a range of *C*_i_ where *J*_PSII_ is at a plateau is indicative of *A*_sat_ being limited by the capacity to regenerate RuBP in the Calvin cycle (Long and Bernacchi, 2003). For both *35S*::*ATHB17* lines, *J*_max_ estimated from these three data points was significantly higher than for the control line (Table 2). At the three lowest C_i_ values, *A*_sat_ and *J*_PSII_ increased linearly with increasing *C*_i_, observations indicative of *A*_sat_ being limited by the activity of ribulose-1,5-bisphosphate carboxylase oxygenase (Rubisco; *V*_c,max_). For both *35S*::*ATHB17* lines, *V*_c,max_ estimated from these three points was also significantly higher than in the control line (Fig. 6 and Table 2). Stomatal conductance, measured at ambient *C*_a_, was reduced by 17% in the *35S*::*ATHB17* 373 line and by 9% in the *35S*::*ATHB17* 378 line; however, neither effect was statistically significant (*P* >0.2; data not shown).

Much research effort is currently focused on increasing the C3 photosynthetic capacity. Increasing the maximum activation state of Rubisco, the capacity to regenerate RuBP in the Calvin cycle, and conductance of CO_2_ to the sites of carboxylation are all key components of the overall strategy (Long *et al.*, 2006; Zhu *et al.*, 2010; Raines, 2011). This study provides evidence that modulation of *ATHB17* activity can increase the biochemical capacity for photosynthesis in *Arabidopsis*, and could improve leaf photosynthesis at current and predicted future atmospheric CO_2_ concentrations.

### Conclusion

In the QC, ATHB17 appears to be part of a complex negative regulatory loop with other class II HD-Zip genes. Based on microarray data, this regulatory loop controls processes such as auxin transport, embryo development, and axis polarity. Constitutive overexpression of ATHB17, on the other hand, modulates leaf development, producing changes in leaf morphology, plastid number and size, and the capacity for photosynthesis. Such results underscore the utility of functional genomics research to identify candidates for the modification of complex traits in plants.

## Supplementary data

Supplementary data are available at *JXB* online.

Supplementary Fig. S1. Loss-of-function mutants for *ATHB17*.

Supplementary Fig. S2. Expression levels of *ATHB17* in the corresponding T-DNA insertion lines represented in Supplementary Fig. S1.

Supplementary Fig. S3. Quantitative RT-PCR analysis to analyse the differential expression of various genes using RNA isolated from the root tip, quiescent centre (QC), and stele.

Supplementary Fig. S4. When grown under our experimental conditions, *athb17-1* does not produce a visible phenotype.

Supplementary Table S1. Genes found to be significantly regulated, where the Benjamini–Hochberg-corrected *P* value is less than 0.05 and the fold change is greater than 1.3, are shown.

Supplementary Table S2. Genes found to be significantly regulated, which underlie the over-representation presented in Fig. 3, are shown.

Supplementary Data

## Abbreviations:

*C*_t_: cycle threshold
*A*_sat_: rate of light-saturated photosynthesis
*C*_a,_: ambient [CO_2_]
CAT: chloramphenicol acetyltransferase
*C*_i_: substomatal [CO_2_]
DIG: digoxigenin
GO: gene ontology
GUS: β-glucuronidase
HD-Zip: homeodomain-leucine zipper
*J*_PSII_: rate of linear electron transport through photosystem II
MS: Murashige and Skoog
NOS: nopaline synthase
PAR: photosynthetically active radiation
PPF: photosynthetic photon flux
QC: quiescent centre
qRT-PCR: quantitative reverse transcription-PCR
RuBP: ribulose-1,5-bisphosphate
Rubisco: ribulose-1,5-bisphosphate carboxylase oxygenase
SD: standard deviation
SE: standard error
SPAD: soil plant analysis development
UTR: untranslated region
*V*_c,max_: the maximum rate of RuBP-saturated photosynthesis.

## References

[CIT0001] Abramoff MD Magelhaes PJ Ram SJ 2004 Image processing with ImageJ. Biophotonics International 11, 36–42

[CIT0002] Aida M Beis D Heidstra R Willemsen V Blilou I Galinha C Nussaume L Noh YS Amasino R Scheres B 2004 The *PLETHORA* genes mediate patterning of the *Arabidopsis* root stem cell niche. Cell 119, 109–120 15454085 10.1016/j.cell.2004.09.018

[CIT0003] Aldridge C Maple J Moller SG 2005 The molecular biology of plastid division in higher plants. Journal of Experimental Botany 56, 1061–1077 15753112 10.1093/jxb/eri118

[CIT0004] Alonso JM Stepanova AN Leisse TJ 2003 Genome-wide insertional mutagenesis of *Arabidopsis thaliana* . Science 301, 653–657 12893945 10.1126/science.1086391

[CIT0005] Ariel FD Manavella PA Dezar CA Chan RL 2007 The true story of the HD-Zip family. Trends in Plant Science 12, 419–426 17698401 10.1016/j.tplants.2007.08.003

[CIT0006] Baklanov MM Golikova LN Malygin EG 1996 Effect on DNA transcription of nucleotide sequences upstream to T7 promoter. Nucleic Acids Research 24, 3659–3660 8836202 10.1093/nar/24.18.3659 PMC146148

[CIT0007] Bechtold N Jolivet S Voisin R Pelletier G 2003 The endosperm and the embryo of *Arabidopsis thaliana* are independently transformed through infiltration by *Agrobacterium tumefaciens* . Transgenic Research 12, 509–517 12885171 10.1023/a:1024272023966

[CIT0008] Berardini TZ Mundodi S Reiser L 2004 Functional annotation of the *Arabidopsis* genome using controlled vocabularies. Plant Physiology 135, 745–755 15173566 10.1104/pp.104.040071 PMC514112

[CIT0009] Brandt R Salla-Martret M Bou-Torrent J 2012 Genome-wide binding-site analysis of REVOLUTA reveals a link between leaf patterning and light-mediated growth responses. The Plant Journal 72, 31–42 22578006 10.1111/j.1365-313X.2012.05049.x

[CIT0010] Butterfass T 1979 Patterns of chloroplast reproduction. Cell Biology Monographs, Vol. 6 Vienna and New York: Springer Verlag

[CIT0011] Ciarbelli AR Ciolfi A Salvucci S Ruzza V Possenti M Carabelli M Fruscalzo A Sessa G Morelli G Ruberti I 2008 The *Arabidopsis* homeodomain-leucine zipper II gene family: diversity and redundancy. Plant Molecular Biology 68, 465–478 18758690 10.1007/s11103-008-9383-8

[CIT0012] Czechowski T Stitt M Altmann T Udvardi MK Scheible WR 2005 Genome-wide identification and testing of superior reference genes for transcript normalization in *Arabidopsis* . Plant Physiology 139, 5–17 16166256 10.1104/pp.105.063743 PMC1203353

[CIT0013] del Campillo E Abdel-Aziz A Crawford D Patterson SE 2004 Root cap specific expression of an endo-β-1,4-d-glucanase (cellulase): a new marker to study root development in *Arabidopsis* . Plant Molecular Biology 56, 309–323 15604746 10.1007/s11103-004-3380-3

[CIT0014] Doerner P 1998 Root development: quiescent center not so mute after all. Current Biology 8, R42–R44 9427641 10.1016/s0960-9822(98)70030-2

[CIT0015] Elhiti M Stasolla C 2009 Structure and function of homodomain-leucine zipper (HD-Zip) proteins. Plant Signaling and Behavior 4, 86–88 19649178 10.4161/psb.4.2.7692 PMC2637487

[CIT0016] Genty B Briantais JM Baker NR 1989 The relationship between the quantum yield of photosynthetic electron transport and quenching of chlorophyll fluorescence. Biochimica et Biophysica Acta 990, 87–92

[CIT0017] Ha CM Kim GT Kim BC Jun JH Soh MS Ueno Y Machida Y Tsukaya H Nam HG 2003 The *BLADE-ON-PETIOLE 1* gene controls leaf pattern formation through the modulation of meristematic activity in *Arabidopsis* . Development 130, 161–172 12441300 10.1242/dev.00196

[CIT0018] Harris JC Hrmova M Lopato S Langridge P 2011 Modulation of plant growth by HD-Zip class I and II transcription factors in response to environmental stimuli. New Phytologist 190, 823–837 21517872 10.1111/j.1469-8137.2011.03733.x

[CIT0019] Hawker NP Bowman JL 2004 Roles for class III HD-Zip and KANADI genes in *Arabidopsis* root development. Plant Physiology 135, 2261–2270 15286295 10.1104/pp.104.040196 PMC520795

[CIT0020] Hepworth SR Zhang Y McKim S Li X Haughn GW 2005 BLADE-ON-PETIOLE-dependent signaling controls leaf and floral patterning in *Arabidopsis* . Plant Cell 17, 1434–1448 15805484 10.1105/tpc.104.030536 PMC1091766

[CIT0021] Holtan HE Bandong S Marion CM 2011 BBX32, An *Arabidopsis* B-box protein, functions in light signaling by suppressing HY5-regulated gene expression and interacting with STH2. Plant Physiology 156, 2109–2123 21632973 10.1104/pp.111.177139 PMC3149924

[CIT0022] Iacobuzio-Donahue CA Ryu B Hruban RH Kern SE 2002 Exploring the host desmoplastic response to pancreatic carcinoma: gene expression of stromal and neoplastic cells at the site of primary invasion. American Journal of Pathology 160, 91–99 11786403 10.1016/S0002-9440(10)64353-2 PMC1867150

[CIT0023] Izhaki A Bowman JL 2007 KANADI and class III HD-Zip gene families regulate embryo patterning and modulate auxin flow during embryogenesis in *Arabidopsis* . Plant Cell 19, 495–508 17307928 10.1105/tpc.106.047472 PMC1867338

[CIT0024] Jiang K Feldman LJ 2005 Regulation of root apical meristem development. Annual Review of Cell and Developmental Biology 21, 485–509 10.1146/annurev.cellbio.21.122303.114753 16212504

[CIT0025] Jiao Y Tausta SL Gandotra N 2009 A transcriptome atlas of rice cell types uncovers cellular, functional and developmental hierarchies. Nature Genetics 41, 258–263 19122662 10.1038/ng.282

[CIT0026] Kerk NM Ceserani T Tausta SL Sussex IM Nelson TM 2003 Laser capture microdissection of cells from plant tissues. Plant Physiology 132, 27–35 12746508 10.1104/pp.102.018127 PMC1540312

[CIT0027] Kohler RH Hanson MR 2000 Plastid tubules of higher plants are tissue-specific and developmentally regulated. Journal of Cell Science 113, 81–89 10591627 10.1242/jcs.113.1.81

[CIT0028] Lilley CJ Wang D Atkinson HJ Urwin PE 2011 Effective delivery of a nematode-repellent peptide using a root-cap-specific promoter. Plant Biotechnology Journal 9, 151–161 20602721 10.1111/j.1467-7652.2010.00542.x

[CIT0029] Long SP Bernacchi CJ 2003 Gas exchange measurements, what can they tell us about the underlying limitations to photosynthesis? Procedures and sources of error. Journal of Experimental Botany 54, 2393–2401 14512377 10.1093/jxb/erg262

[CIT0030] Long SP Zhu XG Naidu SL Ort DR 2006 Can improvement in photosynthesis increase crop yields? Plant, Cell & Environment 29, 315–330 10.1111/j.1365-3040.2005.01493.x 17080588

[CIT0031] Nawy T Lee JY Colinas J Wang JY Thongrod SC Malamy JE Birnbaum K Benfey PN 2005 Transcriptional profile of the *Arabidopsis* root quiescent center. Plant Cell 17, 1908–1925 15937229 10.1105/tpc.105.031724 PMC1167541

[CIT0032] Ohgishi M Oka A Morelli G Ruberti I Aoyama T 2001 Negative autoregulation of the *Arabidopsis* homeobox gene *ATHB-2* . The Plant Journal 25, 389–398 11260495 10.1046/j.1365-313x.2001.00966.x

[CIT0033] Osteryoung KW McAndrew RS 2001 The plastid division machine. Annual Review of Plant Physiology and Plant Molecular Biology 52, 315–333 10.1146/annurev.arplant.52.1.315 11337401

[CIT0034] Pogson BJ Albrecht V 2011 Genetic dissection of chloroplast biogenesis and development: an overview. Plant Physiology 155, 1545–1551 21330494 10.1104/pp.110.170365 PMC3091115

[CIT0035] Prigge MJ Otsuga D Alonso JM Ecker JR Drews GN Clark SE 2005 class III homeodomain-leucine zipper gene family members have overlapping, antagonistic, and distinct roles in *Arabidopsis* development. Plant Cell 17, 61–76 15598805 10.1105/tpc.104.026161 PMC544490

[CIT0036] Pyke KA 1997 The genetic control of plastid division in higher plants. American Journal of Botany 84, 1017–1027 21708657

[CIT0037] Pyke KA 1999 Plastid division and development. Plant Cell 11, 549–556 10213777 10.1105/tpc.11.4.549 PMC144208

[CIT0038] Pyke KA Leech RM 1991 Rapid image analysis screening procedure for identifying chloroplast number mutants in mesophyll cells of *Arabidopsis thaliana* (L.) Heynh. Plant Physiology 96, 1193–1195 16668319 10.1104/pp.96.4.1193 PMC1080914

[CIT0039] Pyke KA Leech RM 1992 Chloroplast division and expansion is radically altered by nuclear mutations in *Arabidopsis thaliana* . Plant Physiology 99, 1005–1008 16668963 10.1104/pp.99.3.1005 PMC1080576

[CIT0040] R Development Core Team 2007 R: a language and environment for statistical computing. http://www.R-project.org, last accessed 26 July 2013.

[CIT0041] Raines CA 2011 Increasing photosynthetic carbon assimilation in C3 plants to improve crop yield: current and future strategies. Plant Physiology 155, 36–42 21071599 10.1104/pp.110.168559 PMC3075778

[CIT0042] Riechmann JL Heard J Martin G 2000 *Arabidopsis* transcription factors: genome-wide comparative analysis among eukaryotes. Science 290, 2105–2110 11118137 10.1126/science.290.5499.2105

[CIT0043] Ruberti I Sessa G Ciolfi A Possenti M Carabelli M Morelli G 2012 Plant adaptation to dynamically changing environment: the shade avoidance response. Biotechnology Advances 30, 1047–1058 21888962 10.1016/j.biotechadv.2011.08.014

[CIT0044] Sawa S Ohgishi M Goda H Higuchi K Shimada Y Yoshida S Koshiba T 2002 The *HAT2* gene, a member of the HD-Zip gene family, isolated as an auxin inducible gene by DNA microarray screening, affects auxin response in *Arabidopsis* . The Plant Journal 32, 1011–1022 12492842 10.1046/j.1365-313x.2002.01488.x

[CIT0045] Sharkey TD Bernacchi CJ Farquhar GD Singsaas EL 2007 Fitting photosynthetic carbon dioxide response curves for C3 leaves. Plant, Cell & Environment 30, 1035–1040 10.1111/j.1365-3040.2007.01710.x 17661745

[CIT0046] Skuzeski JM Nichols LM Gesteland RF 1990 Analysis of leaky viral translation termination codons *in vivo* by transient expression of improved β-glucuronidase vectors. Plant Molecular Biology 15, 65–79 2103444 10.1007/BF00017725

[CIT0047] Sorin C Salla-Martret M Bou-Torrent J Roig-Villanova I Martinez-Garcia JF 2009 ATHB4, a regulator of shade avoidance, modulates hormone response in *Arabidopsis* seedlings. The Plant Journal 59, 266–277 19392702 10.1111/j.1365-313X.2009.03866.x

[CIT0048] Steindler C Carabelli M Borello U Morelli G Ruberti I 1997 Phytochrome A, phytochrome B and other phytochrome(s) regulate *ATHB-2* gene expression in etiolated and green *Arabidopsis* plants. Plant, Cell & Environment 20, 759–763

[CIT0049] Steindler C Matteucci A Sessa G Weimar T Ohgishi M Aoyama T Morelli G Ruberti I 1999 Shade avoidance responses are mediated by the ATHB-2 HD-zip protein, a negative regulator of gene expression. Development 126, 4235–4245 10477292 10.1242/dev.126.19.4235

[CIT0050] Tiwari S Wang S Hagen G Guilfoyle TJ 2006 Transfection assays with protoplasts containing integrated reporter genes. Methods in Molecular Biology 323, 237–244 16739582 10.1385/1-59745-003-0:237

[CIT0051] Zhu XG Long SP Ort DR 2010 Improving photosynthetic efficiency for greater yield. Annual Review of Plant Biology 61, 235–261 10.1146/annurev-arplant-042809-112206 20192734

