# Supplementary material for: Application of HB17, an Arabidopsis class II homeodomain-leucine zipper transcription factor, to regulate chloroplast number and photosynthetic capacity
Source: J Exp Bot. 2013 Sep 4;64(14):4479–90. doi: 10.1093/jxb/ert261 (PMC3808327; doi:10.1093/jxb/ert261)
Supplement: Supplementary Data [file supp_64_14_4479__index.html]

Application of ATHB17, an Arabidopsis class II homeodomain-leucine zipper transcription factor, to regulate chloroplast number and photosynthetic capacity — Application of HB17, an Arabidopsis class II homeodomain-leucine zipper transcription factor, to regulate chloroplast number and photosynthetic capacity — Supplementary Data 

# Application of HB17, an *Arabidopsis* class II homeodomain-leucine zipper transcription factor, to regulate chloroplast number and photosynthetic capacity

## Supplementary Data

Data files

**Files in this Data Supplement:**

- Supplementary Data - Supplementary Data
- Supplementary Data - Supplementary Data
- Supplementary Data - Supplementary Data
