# Supplementary material for: Application of HB17, an Arabidopsis class II homeodomain-leucine zipper transcription factor, to regulate chloroplast number and photosynthetic capacity
Source: J Exp Bot. 2013 Sep 4;64(14):4479–90. doi: 10.1093/jxb/ert261 (PMC3808327; doi:10.1093/jxb/ert261)
Supplement: Supplementary Data [file supp_ert261_jexbot102095_file003.pdf]

Journal of Experimental Botany

Application of AtHB17, an Arabidopsis class II homeodomain leucine zipper transcription factor, to regulate chloroplast number and photosynthetic capacity

Graham J. Hymus, Suqin Cai, Elizabeth A. Kohl,Hans E. Holtan, Colleen M. Marion, Shiv Tiwari, Don R. Maszle, Marjorie R. Lundgren, Melissa C. Hong, Namitha Channa, Paul Loida, Rebecca Thompson, J. Philip Taylor, Elena Rice, Peter P. Repetti, Oliver J. Ratcliffe, T. Lynne Reuber, Robert A. Creelman

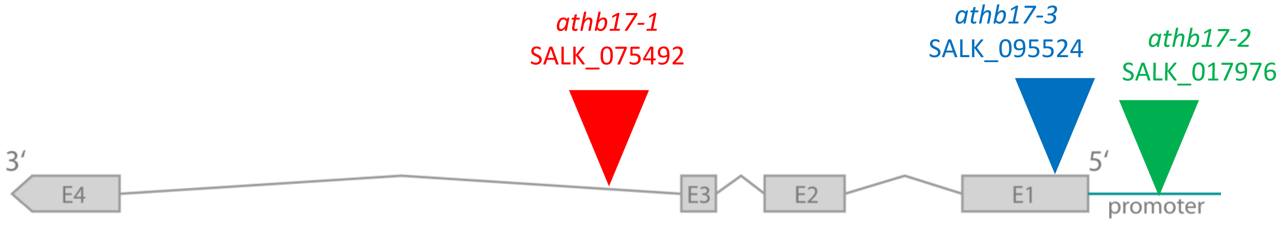

Supplemental Figure 1. Loss-of-function mutants for *ATHB17*. Scheme of the amino terminus of *ATHB17* with location of T-DNA insertions. Database searches revealed the presence of several potential independent T-DNA insertional disruptions of *AtHB17*. The *athb17-1* (SALK_075492), *athb17-2* (SALK_017976) and *athb17-3* (SALK_095524) mutants were obtained from a mutant pool of T-DNA insertion lines deposited into the Arabidopsis Biological Resource Center (ABRC, Ohio State University). To genotype the T-DNA insertions, PCR was performed with primers flanking the T-DNA insertion and primers specific to the T-DNA and adjacent insertion site. The PCR products for *athb17-1*, *athb17-2* and *athb17-3* were sequenced to determine the T-DNA insertion point. Primers used for the T-DNA insertion genotyping were: Salk left border 5'-ATTTTGCCGATTTCGGAAC-3'; *AtHB17* forward 5'- TGGCTCTGCTCCTCCGCGA-3'; *AtHB17* reverse 5'- CCATACCTTTCCCATCTGCTC-3'

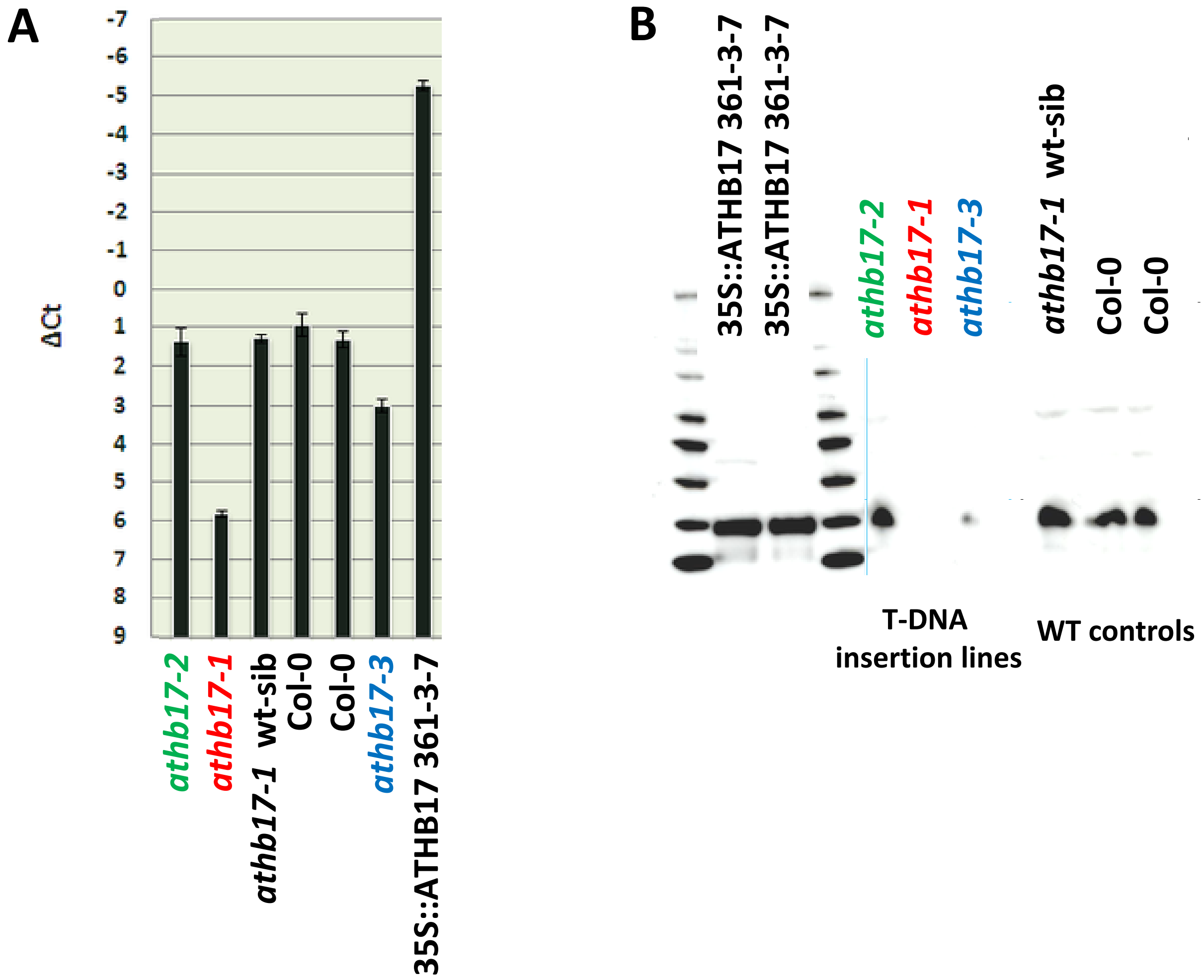

Supplemental Figure 2. Expression levels of *ATHB17* in the corresponding T-DNA insertion lines represented in Supplemental Fig. 1. (A) Total RNA (10 µg) was extracted from the root tips of 11 d old seedlings from the T-DNA insertion lines, the wild type sibling for *athb17-1*, Col-0, and a *35S*::*ATHB17* line. *AtHB17* mRNA levels were quantified by qRT-PCR, and are expressed relative to levels of the constitutive control gene *At2g32170*. AtHB17 protein levels (B) in *athb17-1*, *athb17-2*, *athb17-3*, wild type plants, and an ATHB17 overexpressing line root tips. Approximately 50-fold excess protein compared to that used for the ATHB17 overexpressing line was loaded for lanes corresponding to the T-DNA insertion lines and wild type controls. Soluble proteins were extracted from root tips of 11 d old seedlings of *athb17-1, athb17-2, athb17-3,* and various controls using 50mM Tris pH 7.5, 0.5M NaCl, 1% SDS, 10mM DTT and 2x proteinase inhibitors (Sigma) and then separated on a SDS polyacrylamide gel (4-12%; Novex). Proteins were then transferred to 0.45µm PVDF using XCell II Blot Module (Invitrogen Corporation, Carlsbad CA) according to the manufacturer's specifications. A primary polyclonal antibody raised against a truncated AtHB17(Δ1-73) protein (Epitomics, Inc., Burlingame, CA) was used. The primary antibody was detected using chemiluminescence (WesternBreeze, Novex).

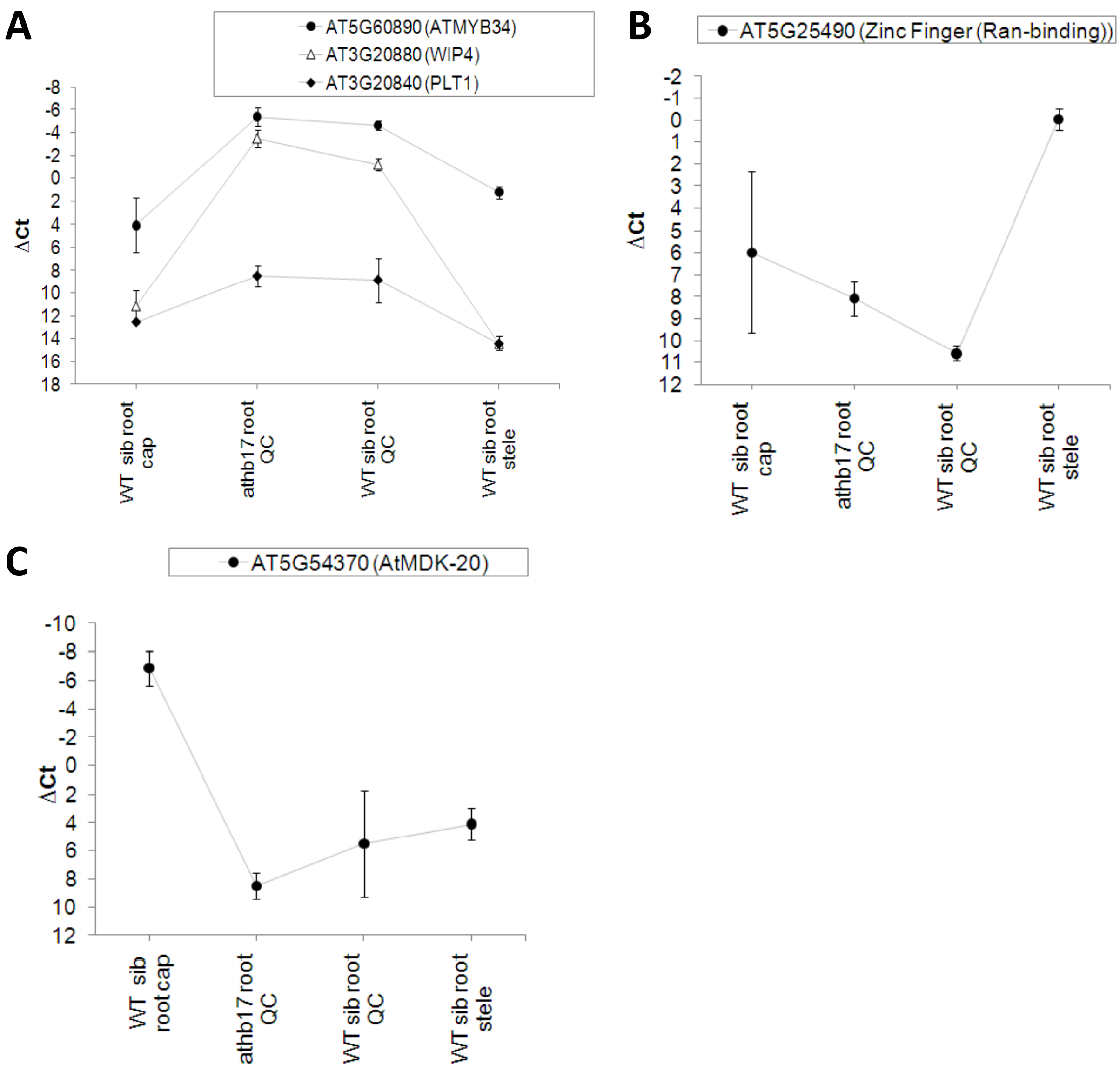

Supplemental Figure 3. Quantitative RT-PCR analysis to analyze the differential expression of various genes using RNA isolated from the root tip, quiescent center (QC) and stele. The expression of PLETHORA 1 (PLT1), WIP4 and ATMYB34 (A) were several fold higher in the QC relative to other root tissues (Nawy et al., 2005). AT5G25490, predicted to encode zinc finger (Ran-binding) family protein, is more abundantly expressed in stele tissue (B). The expression of ATMDK-20 shows root cap enriched expression (C).

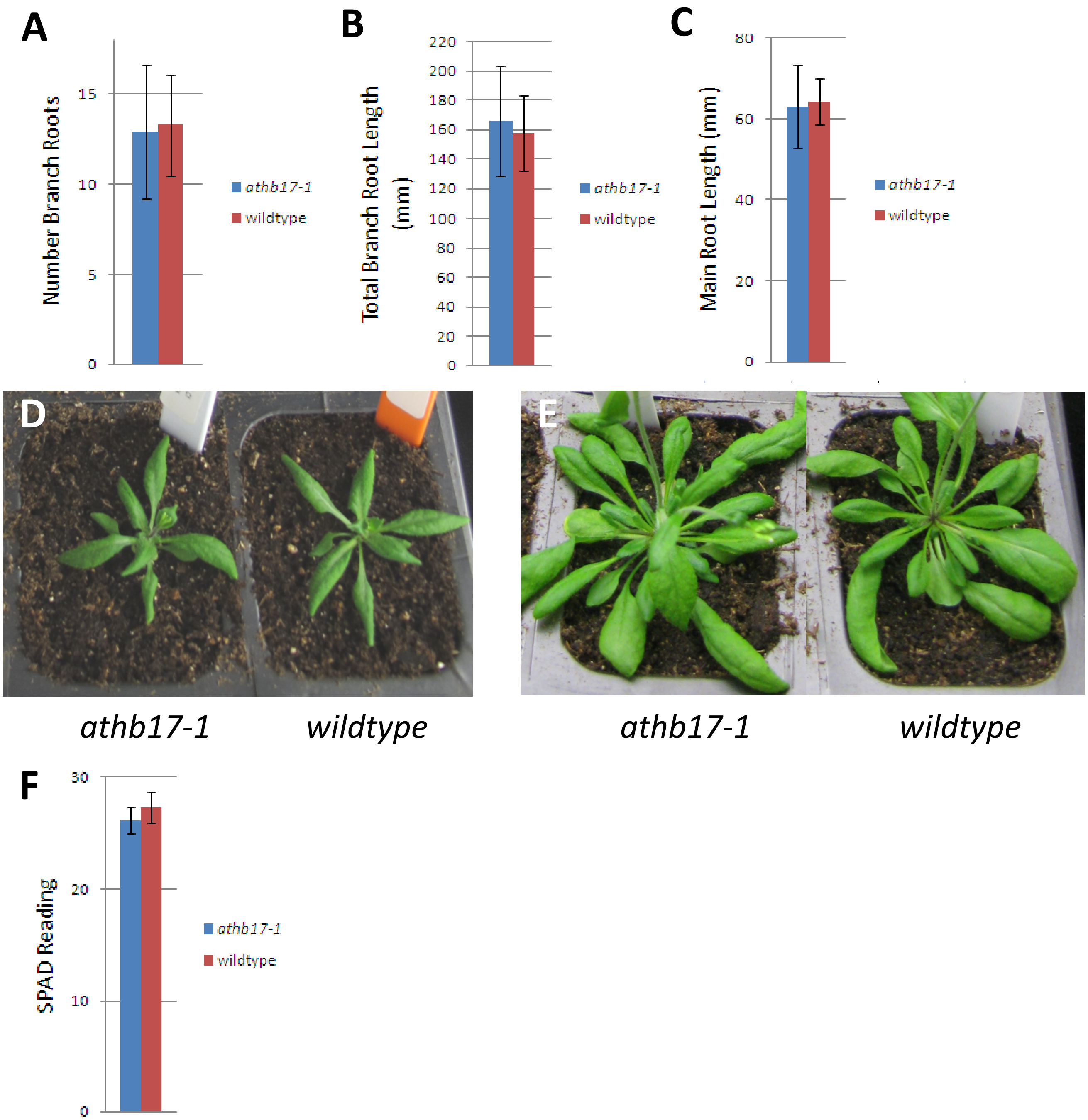

Supplemental Figure 4. When grown under our experimental conditions, *athb17-1* did not produce a visible phenotype. To determine branch root number (A), branch root length (B), and main root length (C) we grew *athb17-1* and its wild-type segregant horizontally on circular plates for 4 days. On day 4, *athb17-1* and its wild-type segregant were transplanted to square plates and grown vertically for 16 days. Root images (n = 14) were traced by hand on the Wacom Cintiq LCD tablet, skeletonized, and analyzed by ImageJ using the Skeletonize3D and AnalyzeSkeleton plugins. Shown is the mean ± standard deviation. To determine rosette phenotypes, we grew *athb17-1* (n = 26 plants) and wild-type control plants on soil under constant light as described in the Materials and Methods. Plants were examined visually for phenotypes at 22 (D) and 30 (E) days after sowing. Chlorophyll content (F) as measured using a Minolta SPAD 502 meter (n =10). Shown is the mean ± standard deviation.
